# Alloactivation of Naïve CD4^+^CD8^−^CD25^+^T Regulatory Cells: Expression of CD8α Identifies Potent Suppressor Cells That Can Promote Transplant Tolerance Induction

**DOI:** 10.3389/fimmu.2019.02397

**Published:** 2019-10-14

**Authors:** Nirupama D. Verma, Catherine M. Robinson, Nicole Carter, Paul Wilcox, Giang T. Tran, Chaunmin Wang, Alexandra Sharland, Masaru Nomura, Karren M. Plain, G. Alexander Bishop, Suzanne J. Hodgkinson, Bruce M. Hall

**Affiliations:** ^1^Immune Tolerance Laboratory, South Western Clinical School of Medicine, UNSW Sydney and Ingham Institute, Liverpool Hospital, Liverpool, NSW, Australia; ^2^Transplantation Immunobiology Research Group, Faculty of Medicine and Health, Charles Perkins Centre, Central Clinical School, University of Sydney, Sydney, NSW, Australia; ^3^Department of Surgery, Keiwakai Ebetsu Hospital, Ebetsu, Japan

**Keywords:** transplant tolerance, T regulatoy cells, antigen specific Treg, CD8α, CD4^+^CD25^+^Foxp3^+^Treg

## Abstract

Therapy with alloantigen-specific CD4^+^CD25^+^ T regulatory cells (Treg) for induction of transplant tolerance is desirable, as naïve thymic Treg (tTreg) are not alloantigen-specific and are weak suppressor cells. Naïve tTreg from DA rats cultured with fully allogeneic PVG stimulator cells in the presence of rIL-2 express IFN-gamma receptor (IFNGR) and IL-12 receptor beta2 (IL-12Rβ2) and are more potent alloantigen-specific regulators that we call Ts1 cells. This study examined additional markers that could identify the activated alloantigen-specific Treg as a subpopulation within the CD4^+^CD25^+^Foxp3^+^Treg. After culture of naïve DA CD4^+^CD8^−^CD25^+^T cells with rIL-2 and PVG alloantigen, or rIL-2 without alloantigen, CD8α was expressed on 10–20% and CD8β on <5% of these cells. These cells expressed *ifngr* and *Il12rb2*. CD8α^+^ cells had increased *Ifngr* that characterizes Ts1 cells as well was *Irf4*, a transcription factor induced by TCR activation. Proliferation induced by re-culture with rIL-12 and alloantigen was greater with CD4^+^CD8α^+^CD25^+^Treg consistent with the CD8α^+^ cells expressing IL-12R. In MLC, the CD8α^+^ fraction suppressed responses against allogeneic stimulators more than the mixed Ts1 population, whereas the CD4^+^CD8^−^CD25^+^T cells were less potent. In an adoptive transfer assay, rIL-2 and alloantigen activated Treg suppress rejection at a ratio of 1:10 with naïve effector cells, whereas alloantigen and rIL-2 activated tTreg depleted of the CD8α^+^ cells were much less effective. This study demonstrated that expression of CD8α by rIL-2 and alloantigen activation of CD4^+^CD8^−^CD25^+^Foxp3^+^T cells was a marker of activated and potent Treg that included alloantigen-specific Treg.

## Introduction

Induction of tolerance to transplanted tissue is a highly desirable goal that has been achieved in a limited number of clinical protocols, reviewed (1). Multiple potential mechanisms may underpin alloantigen-specific transplant tolerance, while T regulatory cells (Treg) contribute tolerance induction and maintenance, reviewed (2). In adult animals made tolerant to an allograft, alloantigen-specific T cells maintain tolerance (3–6) without clonal deletion of alloreactive T cells (7, 8).

Suppressor T cells were first identified in the early 1970's (9) and characterized as CD8^+^I-J^+^ T cells (10, 11). Such suppressor T cells fell into disrepute as the MHC region did not have a gene for I-J, a key marker of the CD8^+^T suppressor cells (12). In transplant tolerance, CD4^+^ not CD8^+^T cells transfer alloantigen-specific tolerance (5, 8, 13) while retaining the capacity to effect third-party rejection (5, 6, 13–15). In some models, CD8^+^T cells can transfer tolerance, however (16–21). Transfer of alloantigen-specific tolerance is dependent upon short-lived cells expressing CD25, the IL-2 receptor alpha chain (8, 13, 15, 22). Alloantigen-specific CD4^+^CD25^+^Treg also mediate tolerance to autoantigens, tumors, and infectious agents (23).

The majority of CD4^+^CD25^+^Treg in peripheral lymphoid tissues are naïve thymus derived Treg (tTreg) that express Foxp3, the transcription factor that induces expression of CD25 and inhibits IL-2 transcription (23). tTreg inhibit antigen presentation, but need ratios of >1:1 to effector linage CD4^+^CD25^−^Foxp3^−^T cells (24–26) to completely suppress allograft rejection (25) and graft vs. host disease (27).

*In vivo*, there is tight homeostatic control of the ratio of CD4^+^CD25^+^Treg to CD4^+^CD25^−^T cells, which is <1:10 even in animals with transplant tolerance (28). Alloantigen-specific Treg in hosts tolerant to an allograft suppress specific donor graft rejection at ratios of <1:10 (15).

For induction of transplant tolerance to haematopoietic stem cells and organ allografts, many groups expand tTreg *in vitro*. The most common method of tTreg expansion uses culture with rIL-2 and anti-CD3 mAb with or without anti-CD28 mAb. Expansion of over 10,000-fold has been reported (29) but these cells are not alloantigen-specific and only fully suppress alloimmune responses at ratios of >1:1 (29). Some early clinical transplant trials have used alloantigen-specific Treg activated by specific alloantigen and rIL-2 (30–33). While these studies suggest the cells are safe, their efficacy is yet to be established.

Our group has focused on producing antigen-specific Treg from naïve tTreg with the hope these will have stable expression of Foxp3 and will retain their regulatory phenotype. We have shown that potent antigen-specific Treg can be induced by activation of tTreg with either allo- or autoantigen (33, 34). Short-term culture of naïve CD4^+^CD25^+^T cells with either rIL-2 or rIL-4 and alloantigen for 3–4 days induces alloantigen-specific Treg (33). These Treg inhibit specific donor but not third-party graft rejection at ratios of ≤1:10 (33). They also have enhanced capacity to inhibit mixed lymphocyte culture (MLC) responses to specific alloantigen (33). rIL-2 and alloantigen activated tTreg induces expression of receptors for type 1 cytokines; interferon-gamma receptor (IFNGR) and rIL-12 receptor beta-2 (IL-12Rβ2) and reduced IFN-γ expression (33). The induction of these receptors was not observed by culture with alloantigen alone. These rIL-2 alloactivated tTreg can be further activated by alloantigen and rIL-12 to highly potent alloantigen-specific Th1-like Treg that suppress fully allogeneic graft rejection in normal hosts with no other immunosuppression (35). In contrast, culture of tTreg with IL-4 and alloantigen induces IL-5Rα, but not IFNGR and IL-12Rβ2 (33). Respectively, we named these lineages Ts1 and Ts2 as Treg that were respectively induced by Type-1 and Type-2 cytokines (33).

For expansion of tTreg with specificity to alloantigen that could be used to induce tolerance *in vivo*, it is highly desirable to identify a way to select the Treg activated by alloantigen from those that do not recognize specific alloantigen and are polyclonally expanded. In this study, we sought additional markers that may distinguish the alloantigen-specific Treg from those polyclonally expanded by rIL-2. After 4 days in culture with rIL-2 alone or with alloantigen, 7–27% of cells were CD4^+^CD8^+^CD25^+^T cells whereas the original freshly enriched naïve Treg population was >98% CD4^+^CD8^−^CD25^+^T cells and had <2% CD4^+^CD8^+^CD25^+^T cells. They also expressed IFNGR and responded to rIL-12. We demonstrated that the cells that *de novo* expressed CD8α included alloantigen-specific Treg that induced transplant tolerance and suppressed alloimmunity in MLC. This finding may allow identification of IL-2 activated Treg that can be activated by an antigen to produce and expand antigen-specific Treg for use as immunotherapy.

## Materials and Methods

### Animals

DA (RT1^a^), PVG (RT1^c^), and Lewis (RT-1^l^) rats were bred and maintained in the animal house, Liverpool Hospital. All animals were fed standard chow and given water *ad libitum*. The study was carried out in accordance of the “Australian Code for the Care and Use of Animals for Scientific Purposes (NHMRC)” and was approved by the Animal Ethics Committee of UNSW, Sydney, NSW, Australia.

### Monoclonal Antibodies (mAb)

Anti-rat mAb specificity-flurochrome (clone number) used for immunostaining were CD4-PE-Cy5 (W3/25), CD8alpha-FITC or PerCP-Cy5.5 (MRC Ox8), CD8beta-FITC (341), CD25/IL-2R alpha-PE (MRC Ox39), MHC class II-FITC (MRC Ox6), CD62L (L-selectin)-FITC (HRLI), and CD45RA-FITC (MRCOx33); all from BD-PharMingen, San Diego, CA and anti-mouse/rat Foxp3-FITC (FJK16s) from eBioscience, San Diego, CA. For dead cell exclusion fixable violet dye FVS660 (BD) was used.

### Cell Preparation and Immunostaining

Spleen and lymph nodes (cervical and mesenteric) from naïve DA rats were harvested and single cell suspension prepared as described (36, 37). RBCs were lysed from single cell suspensions from spleen using lysis buffer (Biolegend). Cell suspensions from spleen and lymph nodes were combined and cells were resuspended in PBS/0.4% BSA (MultiGel, Biosciences, Castle Hill, NSW, Australia). Cells were subjected to immunostaining using mAb against lymphocyte markers and subjected to enrichment of lymphocyte subsets.

Flow cytometry data was acquired on a FACScan or FACS Canto II (Becton Dickinson, San Jose, CA) using CellQuest or FACS DIVA (BD), as described (28, 35). In some experiments doublets were excluded before cells in lymphocyte gate were subjected to dead cell exclusion and live cells were analyzed for expression of cell surface markers. Foxp3^+^ cells analysis did not include a live cell gate, as the dead cell marker and Foxp3 were both on the APC channel.

### Cytokines

Cytokines were produced from transfected cell lines for rIL-2 and rIL-12 with the assistance of Dr. X. Y. He (38) and were quantified, as described (35, 38). Each cytokine was added to cultures at ≥200 units/ml.

### Enrichment of Lymphocyte Subsets

An indirect panning technique was used to deplete CD8^+^T and B cells, as described (36). Briefly, cells were incubated with optimized concentrations of MRC Ox8 and MRC Ox33 (binds B cells and other cells but not T cells), washed with PBS/0.4%BSA then resuspended at 2 × 10^7^ cells/ml. Cells were then incubated on Petri dishes (Greiner βio-one, Kremsmuenster, Austria) coated with rabbit anti-mouse Ig and rabbit anti-rat Ig (Dako A/s. Glostrup, Denmark). This obtained 95–99% enrichment for CD4^+^T cells.

The enriched CD4^+^T cells were incubated at 4°C for 20 min with PE conjugated MRCOx39, then washed twice before incubation for 15 min at 4°C with 8μ*l* cells of mouse anti-PE microbeads (Miltenyi)/ 10^6^ cells, as described (24, 25). The cells were washed to remove unbound beads and were applied to a LS MACS column (Miltenyi) as per manufacturer's instruction. The positively selected CD25^+^ population was resuspended either in media with 20% Lewis rat serum for use in cultures or in PBS/0.2%BSA for injection to rats. The enriched CD4^+^*CD*25^−^T cells were >96% CD4^+^ with <3% CD25^hi^ cells and were used as naïve responder cells in suppressor MLC assay. The enriched CD4^+^*CD*25^+^T cells were 50–80% Foxp3^+^ and 85–95% CD25^+^ with greatest enrichment for CD25^hi^ cells. Most preparations of CD4^+^*CD*25^+^T cells had <1% CD8α^+^ cells, never more than 2% CD8α^+^ cells.

To enrich CD8α^+^ cells post-culture, the unfractionated Ts1 cells from 3 to 4 days culture, having both CD4^+^CD8^+^CD25^+^T and CD4^+^CD8^−^CD25^+^T cells, were incubated with MRC Ox8 to enrich the CD8α^+^ fraction from the CD8^−^ cells using a panning method as described (36). The supernatant containing CD4^+^CD25^+^CD8^−^T cells was collected, and the bound cells were collected to produce a CD4^+^CD25^+^CD8α^+^T cell population.

### Mixed Lymphocyte Cultures to Activate Alloantigen-Specific Treg

All experiments used CD4^+^CD25^+^ cells from naïve DA rats as responder cells and either PVG or Lewis thymic stimulator cells, as described (24, 28, 33, 35). The cell culture medium used was RPMI 1640 (GIBCO, Grand Island, NY) supplemented with 100 ng/ml penicillin, 100 U/ml streptomycin (Glaxo, Boronia, Victoria, Australia), 2 mM L-glutamine, 5 × 10^5^ M 2-mercaptoethanol (Sigma Chemicals, St. Louis, MO), and 20% Lewis rat serum.

Stimulator cells were irradiated (25 Gy) cells from thymus of naïve PVG or Lewis rats (39). Thymic stimulator cells do not produce cytokines and 10^4^ of these stimulators are as effective at stimulation as 2 × 10^5^ *in vitro* irradiated spleen cells (24).

As naïve CD4^+^CD25^+^T cells proliferate poorly in MLC without rIL-2, the methods were refined to eliminate non-specific background proliferation, as described (24, 33, 35). In particular, Lewis rat serum with a low induction of proliferation was used rather than xeno-sera.

For bulk cultures, 2 × 10^6^ naïve CD4^+^CD25^+^T cells were cultured with 10^6^ stimulator cells in 25 cm^2^ flasks (Griener) for 4 days. Medium was supplemented with either rIL-2 (200 units /ml) or rIL-12 (200 units/ml). Cells were cultured at 37°C in humidified air containing 5% CO_2_ and for preparation of Ts1 cells, the cultures were harvested at day 4.

### Suppressor Mixed Lymphocyte Culture Assays

Cultures in U-bottom micro titer plates (Linbro, Flow Labs, VA) had 2 × 10^4^ stimulators cells and either 2 × 10^5^ or 1 × 10^5^ responder cells/well in a total volume of 200 μl. The Treg population was added in serial 2-fold dilutions to give ratios of 1:2 to 1:1024 to CD4^+^CD25^−^ effector cells. Four to six replicate wells were set up for each experimental sample.

Cells were cultured at 37°C in humidified air containing 5% CO_2_ and at various time points, usually at day 4, 5, and 6, the cultures were pulsed with 0.5 μCi ^3^H-TdR (Amersham, Arlington Heights, IL) 16 h prior to harvesting with a Tomtec Cell Harvester 96 Mach IIIM (Tomtec, Hamden, CT). Proliferation was assayed by adding liquid scintillation fluid before counting on a beta counter (1450 Microbeta Plus, Beckman Instruments, Palo Alto, CA). Percentage suppression was calculated using the formula;

(1) $$\begin{array}{l} \%\text{ Suppression}=\frac{\text{Teff prolif with Ag alone }-\text{ Teff prolif with Ag and Treg}}{\text{Teff prolif with Ag}}\;\\ \;\times \;100 \end{array}$$

### RT-PCR

RNA extraction, cDNA synthesis and semiquantitative PCR were performed as described (33, 35, 39). Previously described primers were used for *Gapdh, Il2, Ifng, Il5, Il10, Tnfa, Prf1* (40); *Ifngr, Il5ra, Foxp3, Tbet* and *Gata3*^33^, *Gzmb*,^40^ *Il17*^34^ and *Il12rb2*^35^. Primers for *Irf4* were F-TGTCCTCCGTGAGCTGTCTG R- CCTGGATCGGCTCCTCTATG.

Real time RT-PCR was performed on a Rotorgene (Corbett Research, Mortlake, NSW, Australia) using SYBR Green I and HotMaster Taq polymerase (Eppendorf AG, Hamburg, Germany) or SensiMix DNA kit (Quantace). Gene copy number was derived from a standard curve run in parallel and was normalized against GAPDH expression.

### Operative Procedures

DA rats weighing 200 to 250 gm were anesthetized with either ether or isoflurane and heterotopically grafted with an adult PVG heart, as described (36). Graft rejection was monitored by palpation of beat, daily for 2 weeks then every second day. Graft function was scored using a semi-quantitative scale as described (15, 25). Briefly, ++++ was a full fast beat and no graft dysfunction, +++ some slowing and minor swelling of the graft, ++ significant swelling and slow beat, + very weak beat and marked swelling, 0 no palpable beat and markedly swollen heart.

### Adoptive Transfer Assay

DA rats were irradiated with 7 Gy at the Liverpool Hospital Radiation Oncology Unit the day before heart grafting as previously described (15, 25). This irradiation ablates graft rejection until animals are restored with 5 × 10^6^ naïve CD4^+^T cells, which restores graft rejection (5, 25, 36). To test the capacity of *in vitro* activated Treg to suppress, 0.5 × 10^6^ of these cells were co-administered with the 5 × 10^6^ naïve CD4^+^T cells. Some data from control groups has been previously published (33). At this ratio of 1:10, fresh naïve CD4^+^CD25^+^Treg do not prevent rejection nor induce tolerance (25, 33). Naïve CD4^+^CD25^+^Treg that have been cultured with PVG stimulators and 200 units/ml of rat rIL-2 for 3 days (unfractionated Ts1), when given at a ratio of 1:10 with naïve CD4^+^T cells, suppress PVG but not third-party Lewis heart graft rejection (33).

Adoptive hosts, 40 days after transplantation, had their lymph node and spleen cells harvested. Cells were isolated, stained for subset analysis and the enriched CD25^+^T cells were subjected for FACS and RT-PCR studies. Donor and recipient hearts were examined by routine histology with haematoxylin and eosin (H&E) staining.

### Statistical Analyses

Parametric data were expressed as mean ± standard deviation. Means were compared using Student′s *t*-test using Statview (Abacus Concepts, Berkley, CA) program for Apple Macintosh. Graft survival was non-parametric and was compared with a rank sum test. Statistical significance was set at *p* < 0.05.

## Results

### Naïve CD4^+^CD8^−^CD25^+^Treg Cultured With rIL-2 With or Without Alloantigen Show an Increase in the Proportion of Cells That Expressed CD8 Alpha

**Figure 1:**
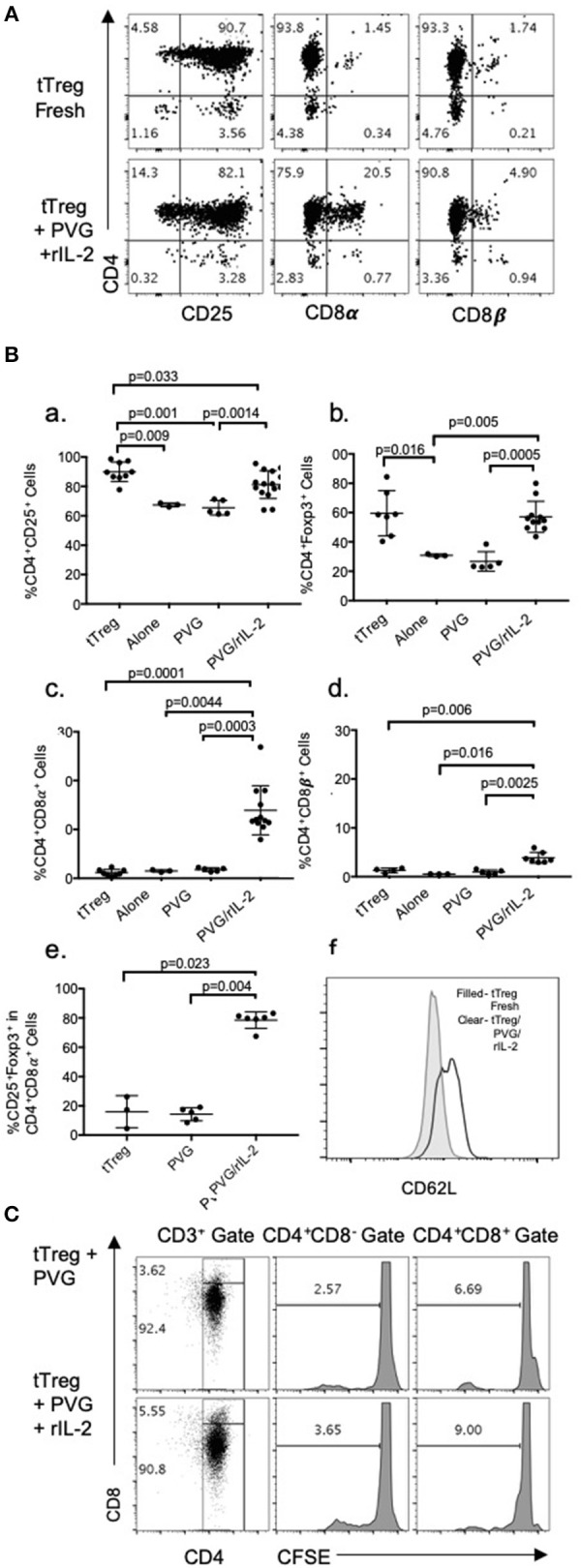
**(A)** FACS profile of freshly isolated CD4^+^CD25^+^T cells (tTreg) from naïve DA rats (Top row) showed >90% cells expressed CD4 and CD25 and <2% expressed either CD8α of CD8β. Bottom row shows tTreg cultured with rIL-2 and PVG alloantigen (Ts1); >80% expressed CD25, about 20% were CD8α^+^ and 5% CD8β^+^. This was consistent with expression of CD8α homodimers. **(B)** Comparison of phenotype of fresh naive tTreg, to these cells after culture for 4 days in medium alone, with PVG alloantigen or with PVG alloantigen and rIL-2. Data from 4 to 5 repeat experiments shown with significant differences if *p* < 0.05 as determined in a Student *t*-test. Culture with PVG and rIL-2, preserved the proportion of CD4^+^CD25^+^Foxp3^+^ cells. After culture with media alone or PVG alone there was a reduction in CD4^+^CD25^+^cells (a) and CD4^+^Foxp3^+^ cells which fell to about 30% (b), consistent with tTreg not surviving without rIL-2. CD4^+^CD8α^+^ cells were increased only after culture with PVG and rIL-2 and represented 10–20% of cells (c), whereas there was a lesser increase in CD4^+^CD8β^+^T cells (d). Thus, there was mainly induction of CD8α homodimer and less CD8αβ heterodimer. The CD4^+^CD8α^+^T cells were mainly CD4^+^Foxp3^+^ cells (e) suggesting they were regulatory T cells. (f) Compared to fresh naïve tTreg the expression of CD62L increased after their culture with rIL-2 and alloantigen (Data representative of 4 experiments). **(C)** tTreg proliferation with rIL-2 and alloantigen: Freshly isolated naïve DA CD4^+^CD25^+^T cells were labeled with CFSE and cultured for 4 days with PVG alone or in presence of rIL-2. Cells were stained with CD4/CD8α/CD3 mAb and gated as CD4^+^CD8^−^ or CD4^+^CD8α^+^. CFSE dilution assessed on a FACS Canto showed proliferation was greater in the CD8α^+^ cells.

Enriched CD4^+^CD8^−^CD25^+^Treg were prepared from lymph node and spleen cells of naïve DA rats by depletion of CD8α^+^ and B cells followed by positive selection of CD25^+^ cells with an anti-CD25 mAb to produce fresh tTreg (Figure 1A). The enriched naïve CD4^+^CD25^+^T cells included <2% (usually <1%) CD8α^+^ cells and <5% CD25^−^ cells (Figures 1A,B). Over 90% had high to intermediate expression of CD25. After culture with PVG alloantigen and rIL-2 to induce Ts1 cells, >80% had intermediate or high expression of CD25, and <4% were CD4^−^.

Culture of these naïve tTreg cells with rIL-2 and PVG alloantigen resulted in a phenotype change with between 8 and 28% expressing CD8α, which was significantly greater than fresh tTreg or tTreg cultured alone or with alloantigen (Figure 1Bc). A smaller proportion, about 5%, expressed CD8β (Figures 1A,Bd). Thus, the majority of CD4^+^CD25^+^Foxp3^+^ cells that expressed CD8α, had CD8αα homodimers and a significant but smaller population were also CD8β^+^ and were CD8αβ heterodimers.

Data from replicate experiments showed culture of tTreg with rIL-2 and alloantigen resulted in a small reduction in the proportion of CD25^+^ cells (Figure 1Ba) but the proportion of Foxp3^+^ cells (~60–70% CD4^+^Foxp3^+^) was maintained (Figure 1Bb). Conversely, control cultures with alloantigen alone or no alloantigen resulted in a reduction of both CD25^+^ cells (Figure 1Ba) and an even greater reduction in Foxp3^+^ cells (Figure 1Bb). Thus, alloantigen stimulation combined with rIL-2 preserved the CD4^+^CD25^+^ population and the majority of these cells continued to express Foxp3.

**Figure 2:**
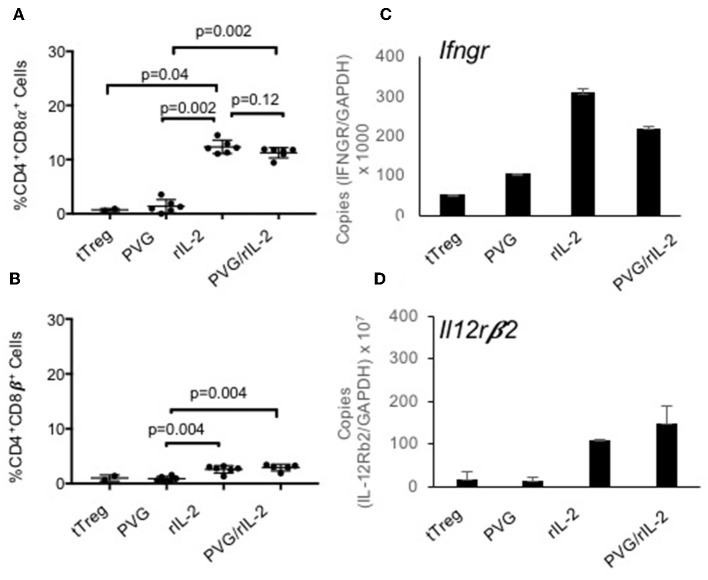
Examination of the effects of culture of naïve CD4^+^CD8^−^CD25^+^Treg with rIL-2 alone to culture with rIL-2 and alloantigen. To examine if the effects of culture were due to rIL-2 or alloantigen, we compared the phenotype of Treg after culture with rIL-2 alone, PVG alloantigen alone to that after culture with both rIL-2 and PVG alloantigen. Cultures for 4 days were carried out as described in Figure 1. **(A,B)** Staining for CD8α and CD8β was as for Figure 1. Culture of fresh tTreg with rIL-2 and no alloantigen also induced CD8α expression similar to when tTreg were cultured with rIL-2 and alloantigen. Again, no CD8α was induced by culture with PVG in the absence of rIL-2. There was a modest induction of CD8β with both rIL-2 and a combination of rIL2 and alloantigen stimulation. Data from two separate experiments and P values were calculated using a student *t*-test. **(C,D)** RT-PCR of tTreg after culture with rIL-2, PVG alloantigen and both rIL-2 and PVG alloantigen showed induction of mRNA for *ifngr* and *Il12b2* in both tTreg cells cultured with rIL-2 alone and in tTreg cells cultured with both rIL-2 and PVG alloantigen, but not in tTreg cultured with no rIL-2 and PVG alloantigen.

About 80% of the CD4^+^CD8α^+^CD25^+^T cells expressed Foxp3 (Figure 1Be) whereas the small proportion of CD4^+^CD8α^+^ in fresh tTreg and after culture of tTreg with PVG and no rIL-2 had low (<20%) expression of Foxp3. Control cultures with rIL-2 without alloantigen showed a similar induction of CD8α to Treg in cultures with rIL-2 and alloantigen (Figure 2A). Thus, it is rIL-2, not alloantigen, that induced CD8α expression by a proportion of the tTreg in culture. rIL-2 alloactivated unfractionated Ts1 cells express a Ts1 phenotype of increased *ifngr* and *Il12rb2* with reduced *ifng*^33^. Compared to fresh naïve tTreg and alloantigen activated Treg, rIL-2 alloactivated tTreg (unfractionated Ts1) and rIL-2 alone activated Treg had increased *ifngr* and *Il12b2* (Figure 2B). There was an approximately a 5-fold increase in mRNA for *Ifngr* (*p* < 0.001) than fresh naïve tTreg.

In all subsequent studies, naïve CD4^+^CD25^+^Treg cultured with alloantigen and rIL-2 had >70% Foxp3^+^ cells.

A CFSE dilution assay to assess naïve CD4^+^CD8^−^CD25^+^ Treg cell division upon culture with rIL-2 and alloantigen showed 9% of the CD4^+^CD8α^+^ cells had divided by day 4, whereas <4% of CD4^+^CD8^−^cells had divided. They both proliferated more than controls stimulated with alloantigen with no rIL-2 (Figure 1C). Thus, CD8α expression was induced on cells that had proliferated (9%) but also on cells that had not proliferated. This is consistent with CD8α expression occurring before clonal expansion of the Treg.

The transplant tolerance transferring CD4^+^T cells do not recirculate from blood to lymphoid tissues (3), whereas naïve tTreg do as they express CD62L. When naïve CD4^+^CD8^−^CD25^+^T cells were cultured with rIL-2 and alloantigen, CD62L expression was increased with a doubling of the mean fluorescence channel (MFI) in the Ts1 population. A representative experiment is shown in Figure 1Bf where tTreg had an MFI of 579, which increased to 1182 on Ts1 cells.

As CD4^+^T cells that transfer tolerance express class II MHC (13), we looked for induction of MHC Class II expression by staining with the mAb MRCOx6. 3–11% of tTreg and 5–9% of Ts1 population expressed class II MHC. Thus, class II MHC was not induced. As CD62L and class II MHC were not useful to identify alloantigen-specific Treg, further characterization was confined to the CD4^+^CD8α^+^CD25^+^ cells.

### Enrichment of the CD8α^+^Population of rIL-2 and Alloantigen Activated CD4^+^CD25^+^T Cells (Unfractionated Ts1)

**Figure 3:**
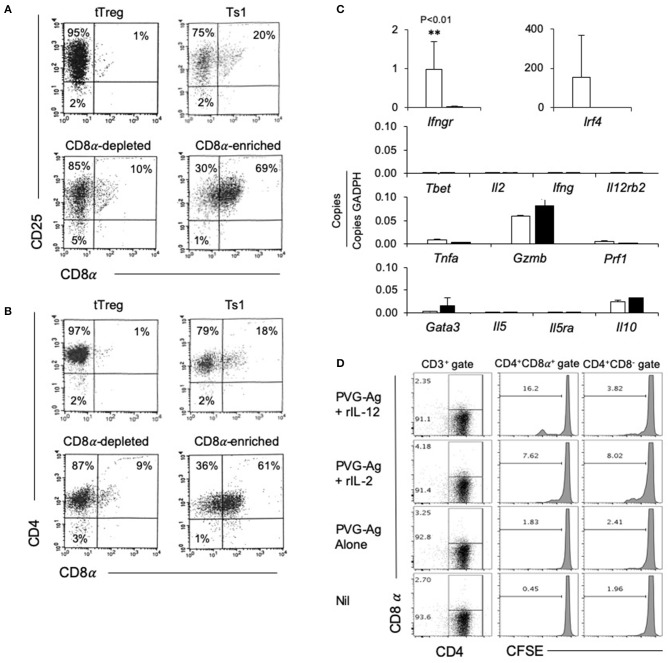
Studies on CD8α-enriched and CD8α-depleted populations prepared from Ts1 cells activated by 4 d culture of CD4^+^CD8^−^CD25^+^T cells from naïve DA rats with rIL-2 and PVG. Freshly isolated CD4^+^CD25^+^T cells (tTreg) from naïve DA rats compared to those cultured with rIL-2 and PVG alloantigen before and after fractionation into CD8α-depleted and CD8α-enriched populations. **(A,B)** FACS profiles of fresh naïve tTreg compared to unfractionated Ts1 cells and the CD8α-enriched and CD8α-depleted populations. All cultured populations had about 95% CD25^+^ cells **(A)** and >95% CD4^+^cells **(B)**. Representative data from one of three experiments. **(C)** RT-PCR of cytokines, cytokine receptors and transcription factors: comparison of expression in CD8α-enriched and CD8α-depleted fractions of Ts1 cells. The CD8α-enriched population (White bars) had significantly higher expression of *Ifngr* (*p* < 0.01) and more *Ifr4* compared to CD8α-depleted fraction (black bars) of Ts1 cells. There was no difference in expression of Type-1 associated molecules *Tbet, Il-2, Ifng, Il12rb2* or cytotoxic cells molecules *Gzmb and Prf1*. There was also no difference in Type-2 associated molecules *gata3, Il5, Il5ra*, and *Il10*. Representative data from one of two experiments. **(D)** Comparison of proliferation of CD8α^+^ and CD8α^−^ cells within Ts1 population further cultured with rIL-2 or rIL-12. Ts1 cells were prepared from naïve tTreg by culturing them with PVG alloantigen and IL-2 for 4 days. Unfractionated Ts1 cells were subsequently cultured either alone, with PVG alloantigen only, and with PVG alloantigen and either rIL-2 or rIL-12 for 3 days. Cells were stained with CD4/CD8α/CD3 antibody and CFSE dilution was assessed on a FACS Canto II to assess proliferation by gating on CD8α^+^ and CD8α^−^ populations within T cells (CD3^+^ Gate). rIL-12 induced more proliferation in the CD8α^+^ cells than in CD8α^−^ cells suggesting these cells expressed IL-12R and responded to this cytokine. Proliferation with rIL-2 was similar in both populations.

The rIL-2 alloactivated Treg (unfractionated Ts1 cells) were separated into CD4^+^CD8^−^CD25^+^T cells and CD4^+^CD8α^+^CD25^+^T cells. Their phenotype was compared to naïve tTreg and the unfractionated Ts1 population (Figures 3A,B). The starting naive tTreg population had 1% CD25^+^CD8α^+^ cells and after culture with rIL-2 and alloantigen, the unfractionated Ts1 cells had 20% CD8α^+^. The CD8α-depleted fraction of Ts1 cells had ≤10% CD8α^+^ cells, mainly low expressing CD8α^+^. The CD8^+^ enriched populations were >60% CD8α^+^ and >99% of CD8α^+^ cells expressed CD25 and CD4.

### Comparison of CD4^+^CD8^−^CD25^+^ and CD4^+^CD8α^+^CD25^+^ Populations of rIL-2 Alloactivated Ts1 Cells for Expression of Cytokine Receptors, Cytokines, and Transcription Factors

We examined the expression of mRNA for various cytokines, transcription factors and cytokine receptors in the CD4^+^CD8^−^CD25^+^ and CD4^+^CD8α^+^CD25^+^ populations of unfractionated Ts1 cells (Figure 3C). The CD8α-enriched (CD4^+^CD8α^+^CD25^+^) population had nearly 40-fold greater *Ifngr* expression (*p* < 0.001) than the CD8α-depleted population of unfractionated Ts1 cells.

The transcription factor *Irf4*, a factor that is induced when antigen activates the specific T cell receptor (TCR), was increased in CD8α-enriched (CD4^+^CD8α^+^CD25^+^) population compared to naïve tTreg but in some experiments *Irf4* was also increased in the CD8α-depleted (CD4^+^CD8^−^CD25^+^) population. Type-1 associated molecules such as *Tbet, Il2, Ifng* were not induced in either population and were not in the starting tTreg population. Effector T cell molecules *Tnfa, Gzmb*, and *Prf1* were similar in both CD8α-enriched and CD8α-depleted Ts1 cells. Type-2 associated molecules *Il5* and *Il5ra* were not expressed, and there was no difference in *Il10*, and *gata3* expression.

We have shown that the receptor for IL-12 (*Il12b2)* is induced on tTreg cultured with rIL-2 and alloantigen and that rIL-12 can induce proliferation of Ts1 cells to specific-alloantigen and induce Th1-like Treg (35). *Il12rb2* levels were too low to assay in both the CD8α^+^ and the CD8α^−^ populations. To assay the functionality of any IL-12R expression, we examined the effect of rIL-12 on proliferation of the CD4^+^CD8α^+^CD25^+^ and CD4^+^CD8^−^CD25^+^ populations of unfractionated Ts1 in the presence of specific alloantigen (Figure 3D). rIL-12 induced greater proliferation in the CD8α^+^ population compared to the CD8^−^ population (16 vs. <4%). rIL-2 induced some proliferation in about 8% of both the CD8^−^ and the CD8α^+^ populations. Proliferation was not induced by re-culture with specific PVG alloantigen alone (<3%) or with no alloantigen or cytokine (<2%). This was consistent with our previous observation that rIL-12 expands the alloantigen-specific population of activated Ts1 cells (35) and suggested that the CD4^+^CD8α^+^CD25^+^Foxp3^+^ T cells include most alloantigen-specific Treg. Only a fraction (approximately one sixth) of the CD8α^+^ cells proliferated showing not all CD8α^+^T cells were alloantigen-specific Treg.

Taken together, these results are consistent with the CD4^+^CD8α^+^CD25^+^ cells containing the activated alloantigen-specific Treg. First, IRF4 is induced by cognate specific antigen activation via the TCR (41). Second, the Ts1 associated cytokine receptor *Ifngr* was expressed mainly in the CD4^+^CD8α^+^ double positive cells. Third, specific alloantigen and rIL-12 induced proliferation of a significant fraction of these double positive cells.

### Comparison of the Ability of CD4^+^CD8^−^CD25^+^ and CD4^+^CD8α^+^CD25^+^ Populations of Ts1 to Suppress Proliferation in an MLC

**Figure 4:**
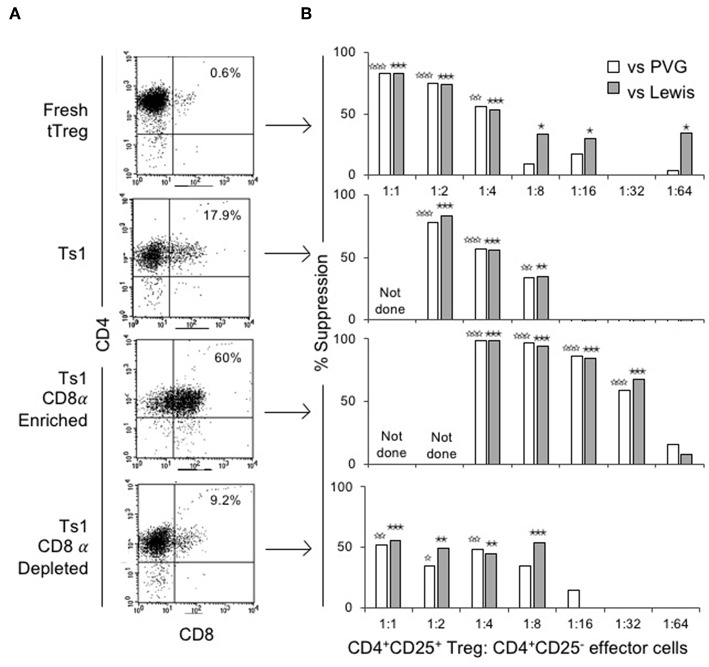
Phenotype and suppression capacity *in vitro* of CD8α expressing population of CD4^+^CD25^+^T cells post-culture with rIL-2 and alloantigen. **(A)** FACS analysis of fresh naïve tTreg pre-culture and post-culture unfractionated Ts1 cells, and CD8α-enriched and CD8α-depleted Ts1 cells. Unfractionated Ts1 had ~18% cells that expressed CD8α, CD8α-enriched cells were 60% CD4^+^CD8α^+^ and CD8α-depleted were only 9% CD4^+^CD8α^+^. **(B)** tTreg, unfractionated Ts1, CD8α-enriched Ts1 and CD8α-depleted Ts1 cells were examined for suppression capacity against CD4^+^CD25^−^T cells from naïve DA rats stimulated in MLC with either specific donor PVG or third-party Lewis alloantigens. A ^3^H thymidine incorporation assay was used to detect proliferation, and cultures were harvested at day 5. Data expressed as percent suppression as: $\%\;\text{suppression}=\;\frac{\text{Teff}\;\text{prolif}\;\text{with}\;\text{Ag - Teff}\;\text{prolif}\;\text{with}\;\text{Ag}\;\text{and}\;\text{Treg}}{\text{Teff}\;\text{prolif}\;\text{with}\;\text{Ag}}X\;100$. All groups had 4–6 replicates. tTreg suppressed up to a ratio of 1:4 while unfractionated Ts1 could suppress up to 1:8 of Treg to T effector cells ratio. CD8α-enriched Ts1 suppressed at 1:32 ratio and CD8α-depleted Ts1 only suppressed up to 1:8 ratio similar to unfractionated Ts1 cells. Significant differences are shown as ☆ (comparison to effector cells proliferation with PVG) or ★ (comparison to effector cells proliferation with Lewis), ^☆^, ^★^*p* ≤ 0.05, ^☆☆^, ^★★^*p* ≤ 0.01, and ^☆☆☆^, ^★★★^*p* ≤ 0.001.

The ability of CD4^+^CD8^−^CD25^+^ and CD4^+^CD8α^+^CD25^+^ fractions of Ts1 cells to suppress proliferation of naïve CD4^+^CD25^−^T cells in an MLC stimulated by either specific donor alloantigen or a third-party alloantigen was compared to fresh tTreg and the unfractionated Ts1 population. Figure 4A shows FACS of the populations used. Fresh tTreg had <1% CD8α^+^ cells compared to 18% in the unfractionated Ts1 population. The CD8α-enriched population had 60% CD8α^+^ cells, although a CD8α^lo^ population contained the majority of cells outside the CD8 gate. The CD8α-depleted population had <10% CD8α^+^ cells with most cells been CD8α^lo^.

Figure 4B shows suppression of proliferation of naïve CD4^+^CD25^−^T effector cells to alloantigen in MLC by serially diluted CD8α^+^ and CD8^−^ populations of Ts1. The CD8α-enriched population of Ts1 suppressed the proliferation of effector cells by >50% out to a 1:32 ratio of CD8α-enriched Ts1 to effector cells. The CD8α-depleted Ts1 cells approached 50% suppression at lower dilution of 1:8, after which there was no suppression. Suppression by the unfractionated Ts1 cells was less than CD8α-enriched population at 1:4 and 1:8 dilution, with no suppression at 1:16 dilution of Ts1 cells to effector T cells. Suppression by all cell populations was not alloantigen-specific as the Ts1 cells activated by PVG suppressed MLC against both PVG and third-party Lewis alloantigen. The data in Figure 4B is expressed as percent suppression for ease of presentation. Proliferation to specific donor with CD8α^+^cells at 1:16 was 1.8 ± 0.4 × 10^3^ cpm and at 1:32 was 5.4 ± 2.3 × 10^3^ cpm, vs. control proliferation with no Treg of 13.7 ± 2.6 × 10^3^ cpm (*p* = 0.003 and *p* = 0.004). Cells depleted of CD8α^+^ cells did not inhibit proliferation at 1:16 (9.6 ± 3.8 × 10^3^ cpm) or at 1:32 (15.0 ± 4.2 × 10^3^ cpm) nor did unfractionated Ts1 cells (data not shown).

### Comparison of the Ability of the CD4^+^CD8^−^CD25^+^ Ts1 Population to the Unfractionated Ts1 Containing Both CD4^+^CD8^−^CD25^+^ and CD4^+^CD8α^+^CD25^+^Cells to Suppress Allograft Rejection Mediated by Naïve CD4^+^T Cells in an Adoptive Transfer Assay

**Figure 5:**
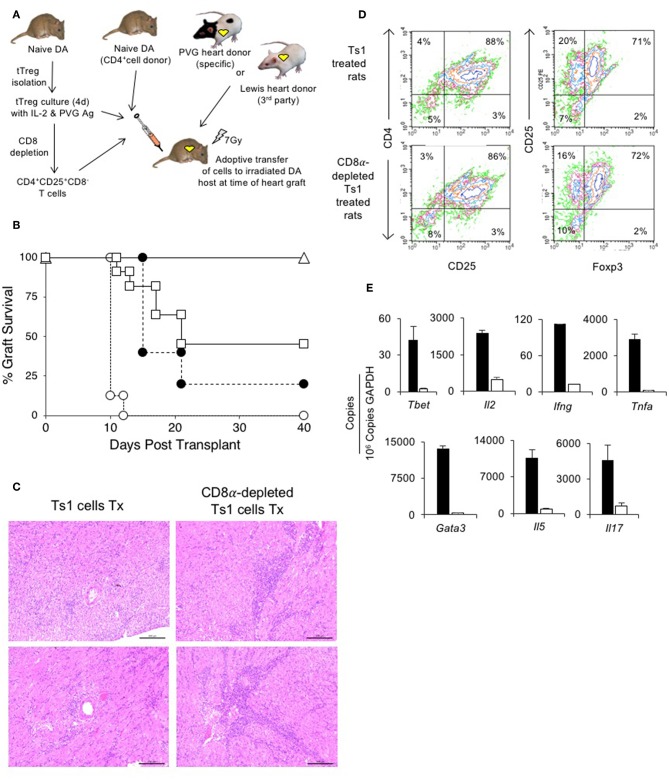
Assessment of suppression by CD8α expressing CD4^+^CD25^+^T cells in an adoptive model of cardiac allograft rejection. **(A)** Scheme of experimental protocol. Irradiated DA hosts were transplanted with PVG heart allograft and given 5 × 10^6^ CD4^+^T cells prepared from naïve DA rats. In this model, irradiated rats do not reject their graft unless reconstituted with naïve CD4^+^T cells that restore rejection in 10–14 days (25). 5 × 10^6^, but not 5 × 10^5^, freshly isolated CD4^+^CD8α^−^CD25^+^ tTreg suppress rejection in hosts restored with 5 × 10^6^ naïve CD4^+^T cells (25). In previous studies, we showed 0.5 × 10^6^ unfractionated Ts1 cells could suppress rejection of specific donor but not third-party Lewis heart grafts (33). To examine role of CD8α^+^Treg in unfractionated Ts1 cell populations, the unfractionated Ts1 cells were produced after culture of naïve tTreg with PVG stimulator cells and rIL-2 for 4 days. CD8α^+^ cells were depleted and the capacity of 5 × 10^5^ CD8α-depleted Ts1 cells to suppress rejection was examined and was compared to that of unfractionated Ts1 cells. **(B)** Survival of PVG cardiac allografts in irradiated DA rats restored with 5 × 10^6^ naïve CD4^+^T cells: effect of CD8α depletion on ability of Ts1 cells to suppress allograft rejection and induce tolerance. 0.5 × 10^6^ unfractionated Ts1 cells suppressed rejection in all hosts with grafts surviving >100 days (Δ) (*n* = 9). This was significantly different to rats that received no Treg and were given 5 × 10^6^ naïve CD4^+^T cells only (O) that rejected their grafts in less than 2 weeks (*n* = 8) (*p* < 0.001). To test the effect of CD8α^+^ cells, Ts1 cells were depleted of CD8α^+^ cells by panning as described in Methods. 0.5 × 10^6^ CD8α-depleted Ts1 cells (□) given with 5 × 10^6^ naïve CD4^+^ T cells (*n* = 11), did not prevent rejection and over halve the animals had rejected their graft within 22 days. This was significantly different to hosts given the same number of unfractionated Ts1 cells that contained CD8α^+^cells (*p* < 0.05), as well as to control rats given no Treg which had more rapid rejection (*p* < 0.001). 0.5 × 10^6^ fresh tTreg (∙) (*n* = 5) delayed rejection compared to controls given no Treg (*p* = 0.05) and their effect was similar to the CD8α-depleted Ts1 cells. These results showed that CD8α^+^ cells in the unfractionated Ts1 cells were required to mediate full suppression of allograft rejection. CD8α^+^-enriched (CD4^+^CD8α^+^CD25^+^T) cells could not be trialed as the anti-CD8 mAb (MRC Ox8) used to enrich these cells opsonizes these cells *in vivo* (44). Results are combined data from four separate experiments. **(C)** Histology of allografts in hosts given unfractionated Ts1 cells or CD8α-depleted Ts1 cells. Photomicrographs of H&E stained cardiac allografts from adoptive hosts 40 days after they were given naïve CD4^+^T cells and either unfractionated Ts1 or CD8α-depleted Ts1 cells. Images taken using a Leica DFC 450C camera on a Leica DM 2000 LED microscope using a 20x magnification. Heart grafts from hosts given CD8α-depleted Ts1 cells had large areas of mononuclear cell infiltration and scattered infiltrate between myocytes. There were wide areas of myocyte necrosis. Heart grafts from animals given unfractionated Ts1 cells had minimal mononuclear infiltration between myocytes and minimal myocyte necrosis. **(D)** FACS profiles of CD25^+^T cells enriched from spleen and lymph nodes of adoptive hosts restored with unfractionated Ts1 cells or CD8α-depleted Ts1 cells. Spleen and LN from adoptive hosts given naïve CD4^+^T cells and either unfractionated Ts1 or CD8α-depleted Ts1 cells were harvested and single cell suspension prepared. Lymphocytes were subjected to single step isolation of CD25^+^T cells. There was no difference in the proportion of CD25^+^ or Foxp3^+^ populations. These cells were used to prepare mRNA for RT-PCR shown in **(E)**. **(E)** RT-PCR of CD25^+^T cells prepared from adoptive hosts restored with naïve CD4^+^T cells and either unfractionated Ts1 cells or CD8α-depleted Ts1 cells. The CD25^+^enriched populations from rats restored with unfractionated Ts1 populations (white bars) and from CD8α-depleted populations (black bars) were subjected to RT-PCR to determine expression of cytokine receptors, transcription factors and cytokines. Cells from hosts that were given CD8α-depleted Ts1 had higher expression of Th1 and Th2 transcription factors, *Tbet* and *Gata3* and cytokine such as *Il2, Ifng, Tnfa, Il17*, and *Il5* consistent with increased inflammation and graft rejection. These findings suggest the CD8α-depleted Ts1 cells were not able to control rejection response and the CD25^+^ cells isolated from these hosts had activated effector cells that expressed mRNA for Th1 and Th2 cytokines.

The adoptive transfer assay reported in this study has been extensively used to study the ability of a cell population to suppress rejection mediated by naïve CD4^+^T cells (5, 13, 15, 25, 42, 43). The assay uses DA rats that had received whole body irradiation (7–8.5 Gy) and had then been grafted with either PVG or Lewis heterotopic heart grafts as illustrated in Figure 5A. Irradiated hosts given no cells do not reject their grafts, which survive over 100 days, whereas the non-irradiated hosts reject their graft in 6–10 days. Reconstitution of the host with peripheral CD4^+^T cells from a normal animal restores rejection to 10–14 days (5, 36, 45). As few as 10^6^ naïve CD4^+^T cells reliably restore rejection. 5 × 10^6^ naïve CD4^+^T cells restored rejection in over 97% of hosts (5, 25, 45).

We have previously reported that 0.5 × 10^6^ unfractionated Ts1 cells activated by culture of fresh naïve CD4^+^CD25^+^T cells with PVG stimulator cells and rIL-2, suppress PVG but not third-party Lewis heart graft rejection in irradiated (7 Gy) DA hosts restored with 5 × 10^6^ naïve CD4^+^T cells^33^. In this study, all 9 irradiated DA rats restored with 0.5 × 10^6^ unfractionated Ts1 cells produced by culture of naïve DA tTreg with rIL-2 and PVG stimulator cells suppress rejection of PVG heart allografts by 5 × 10^6^ co-transferred naïve CD4^+^T cells, with all grafts surviving >40 days (Figure 5B). All animals followed long-term (up to a year) did not reject the allograft. This was significantly different (*p* < 0.001) compared to hosts only given 5 × 10^6^ naïve CD4^+^T cells that all reject in 10–12 days (*n* = 8). This replicates our previous study (33).

To test the specificity of Ts1 cells induced by PVG and rIL-2, we transferred 0.5 × 10^6^ of these cells to irradiated DA rats grafted with third party Lewis heart grafts, and all grafts are rejected in 10 (8–12) days (median (range) (*n* = 6). Controls with third-party Lewis grafts restored with 5 × 10^6^ naïve DA CD4^+^T cells alone reject in 12 (10–>100) days (NSD) (*n* = 7) (33).

Here, we examined if the CD8α^+^ cells in Ts1 cells were required to suppress rejection long-term by depleting CD8α expressing cells from Ts1 (CD4^+^CD8α^−^CD25^+^ Ts1) cells and comparing their ability to suppress allograft rejection with unfractionated Ts1 cells. 0.5 × 10^6^ CD4^+^CD8α^−^CD25^+^ Ts1 cells failed to suppress rejection in 7 of 11 adoptive hosts, significantly different (*p* < 0.01) compared to those given unfractionated Ts1 cells where rejection was suppressed in all 9 animals. The effect of 0.5 × 10^6^ CD4^+^CD8α^−^CD25^+^ Ts1 cells was not different to the effects of 0.5 × 10^6^ naive CD4^+^CD25^+^T cells where 4 of 5 grafts were rejected. Thus, the CD4^+^CD8α^−^CD25^+^ Treg had no increase in potency to suppress responses against specific donor.

The enriched CD4^+^CD8α^+^CD25^+^populations were not directly studied as the mAb, MRC OX 8, used to enrich these cells opsonizes the cells *in vivo* (44). All controls restored with 5 × 10^6^ naïve CD4^+^T cells alone rejected faster than animals given CD4^+^CD25^+^T cells. Only the unfractionated Ts1 cells containing the CD8α^+^ fraction reliably induced tolerance suggesting this population contained the cells whose potency had been increased by activation by rIL-2 and alloantigen.

At 40 days, we compared the PVG heart grafts of host restored with unfractionated Ts1 cells to those given the CD8α-depleted Ts1 population. The grafts given CD8α-depleted Ts1 cells had marked graft destruction and fibrosis with a widespread mononuclear cell infiltrate (Figure 5C). Those given unfractionated Ts1 cells had good graft morphology and a mild mononuclear cell infiltrate with cells scattered between cardiac myocytes.

CD25^+^T cells were enriched from lymph node and spleen cells from adoptive hosts with PVG heart allografts that had been restored with unfractionated Ts1 cells containing both CD4^+^CD8α^+^CD25^+^T cells and CD4^+^CD8^−^CD25^+^T cells and also from those restored with Ts1 cells depleted of the CD8α^+^ subset. Cells were subjected to flow cytometry and mRNA extraction for RT-PCR. Their FACS profiles showed no difference in the proportion of CD4^+^CD25^+^ cells, with just over 70% expressing Foxp3 (Figure 5D).

RT-PCR showed higher expression of inflammatory cytokines and transcriptional factors in CD25^+^T cells from hosts restored with the CD4^+^CD8^−^CD25^+^T cells compared to that from hosts restored with unfractionated Ts1 cells (Figure 5E). There was less mRNA for Th1 associated molecules *Tbet, Il2, Ifng*, and *Tnfa* in CD4^+^CD25^+^ T cells from adoptive host restored with unfractionated Ts1 cells. mRNA for Th2 associated molecules *Gata3* and *Il5* were also less in the tolerated grafts of hosts given unfractionated Ts1 cells, as was mRNA for the Th17 molecule *Il17a*.

## Discussion

There is considerable interest in the expansion and use of Treg for therapy in transplantation and autoimmunity (29). Much work has focused on the expansion of tTreg, usually by polyclonal expansion with rIL-2 combined with anti-CD3 mAb and anti-CD28 mAb but this does not promote expansion of alloantigen-specific Treg. Several groups have shown that culture of tTreg with rIL-2 and alloantigen does induce a more potent alloantigen-specific Treg that suppress alloimmune responses *in vitro* at ratios of 1:32 to 1:64 using both human (30) and murine CD4^+^CD25^+^Treg (33, 35). In the processes of activating tTreg with rIL-2, there are two pathways of activation. First, tTreg with a TCR for specific alloantigen present in the cultures are activated, and these cells can promote alloantigen-specific tolerance. Second, there is polyclonal proliferation of tTreg with TCR that do not recognize the specific alloantigen in culture. A method that selects the Treg activated by rIL-2 and alloantigen from those that do not recognize specific alloantigen would be highly desirable.

The simplest explanation of our results is that on exposure to IL-2, a proportion of CD4^+^CD8^+^CD25^+^Foxp3^+^Treg are induced to express CD8α alone or with CD8β and this step is not dependent upon antigen. Further they express *Ifngr* and *il12rb2*. This antigen non-specific activation step potentially prepares TCR on tTreg to recognize antigen presented by class I MHC as well as class II MHC. It also allows their further activation by Type-1 cytokines, IFNγ and IL-12. We suggest that within the CD8α expressing cell population there are cells with TCR specific for alloantigen that can proliferate to produce more potent antigen specific Treg. The CD4^+^CD8^−^CD25^+^Foxp3^+^Treg in cultures with rIL-2 remain like tTreg with no antigen specificity and no increased potency.

In this study, we identified that the major increase in potency of Ts1 cells activated by the alloantigen and rIL-2 in the culture was by cells that expressed CD8α. This increased proportion of CD4^+^CD8α^+^CD25^+^Foxp3^+^T cells after culture with rIL-2 and alloantigen or with rIL-2 alone, could be from *de novo* induction of CD8α expression by CD4^+^CD8^−^CD25^+^Foxp3^+^ or a selective expansion of the remaining <2% CD8α^+^ cells in the initial tTreg preparation. As many tTreg preparations had <1% CD8α^+^ cells, the induction of *de novo* CD8α expression seems the more probable source to the CD4^+^CD8α^+^CD25^+^Foxp3^+^T cells. Further, only a small fraction of the CD4^+^CD8α^+^CD25^+^Foxp3^+^T cells had proliferated, suggesting CD8α expression occurs before proliferation. Culture of tTreg with rIL-2 alone with no alloantigen also induces CD8α expression on tTreg and increased expression of *ifngr* and *il12rb2*.

In previous studies, we have identified that tTreg cultured with rIL-2 and alloantigen are induced to express receptors for Th1 associated cytokines, IFN-γ and IL-12 (33, 35). In this study, we found it was the CD4^+^CD8α^+^CD25^+^T cells, not the CD4^+^CD8^−^ CD25^+^T cells that expressed *Ifngr*. Although by RT-PCR *Il12rb2* was not detected in either population, when cultured with specific alloantigen and rIL-12, it was only some CD4^+^CD8α^+^CD25^+^T cells that had cells that proliferated, consistent with the alloantigen specific Treg being in this population. It is possible that stimulator cells had some of this IFNGR and IL-12β2 expression, however these cells would be equal in both separated populations. Further, CD4^+^CD8α^+^CD25^+^T cells had more induction of *Irf4*, a transcription factor induced when TCR is activated by antigen. These findings suggested that the tTreg with TCR specific for the alloantigen are part of the subpopulation that is induced to express CD8α, *Ifngr* and *Il12rb2* that we had previously found on unfractionated Ts1 cells (33, 35).

We demonstrated that the CD4^+^CD8α^+^CD25^+^T cells were required to fully suppress allograft rejection and induce transplant tolerance in our adoptive transfer assay. We have previously published that in this assay irradiated hosts do not reject grafts, but reliably do so if restored with 5 × 10^6^ naïve CD4^+^T cells. We also have shown that 0.5 × 10^6^ unfractionated Ts1 cells fully suppress rejection mediated by 5 × 10^6^ naïve CD4^+^T cells of specific donor PVG grafts but not of third-party Lewis grafts (33) demonstrating alloantigen-specific Ts1 cells. The animals given unfractionated populations that contain the CD8α^+^ cells had no rejection at any point post-transplant and all developed tolerance with long-term graft survival. 0.5 × 10^6^ fresh naïve CD4^+^CD25^+^T cells do not fully suppress rejection and do not reliably induce long-term tolerance in these hosts. In this study, we showed that depletion of the CD4^+^CD8α^+^CD25^+^T cells from the rIL-2 and alloantigen activated unfractionated Ts1 cells led to loss of capacity to induce tolerance and the results were similar to naïve tTreg. Both naïve tTreg and CD8α depleted Ts1 populations delay full rejection and, in some animals, induce tolerance. This showed the CD4^+^CD8α^+^CD25^+^T cells in the activated unfractionated Ts1 cells were more effective at mediating alloantigen-specific suppression of graft rejection. We were unable to directly test the effects of the CD4^+^CD8α^+^CD25^+^Treg subset as MRC OX8, the mAb used to enrich this CD8 subset, depletes CD8^+^T cells *in vivo* (44).

One characteristic of rIL-2 and alloantigen activated Ts1 cells is that they can be further activated by rIL-12 and the specific alloantigen to induce Th1-like Treg that suppress proliferation of CD4^+^CD25^−^T effector cells to alloantigen *in vitro* at a ratio of 1:1024 and *in vivo* at a ratio of 1:100 effector T cells (35). These Th1-like Treg are not induced by repeated culture with rIL-2 and alloantigen but are by re-culture with alloantigen and rIL-12 in the absence of rIL-2 (35). In this study, we showed that during re-culture of the unfractionated Ts1 cells with rIL-12 and alloantigen it was the CD4^+^CD8α^+^CD25^+^T cells that proliferated. This was not observed when unfractionated Ts1 cells were re-cultured with rIL-2 and specific alloantigen. On the other hand, the CD4^+^CD8α^−^CD25^+^T cells showed no increase in proliferation when unfractionated Ts1 cells were re-cultured with rIL-12 and alloantigen, but proliferation within this fraction was higher when they were cultured with rIL-2 and specific alloantigen. This suggested that the CD4^+^CD8α^+^CD25^+^Treg were the cells that could be expanded to very suppressive Th1-like Treg whereas the CD4^+^CD8α^−^CD25^+^Treg population could be expanded polyclonally by rIL-2.

The CD8α-enriched fraction of Ts1 cells (CD4^+^CD8α^+^CD25^+^T cells) did not have characteristics of the Th1-like Treg as they did not express *Ifng* or *Tbet*. They also did not express *Il2, Il4, Il5*, and expression of *Foxp3, Perf*, *Gzmb*, or *Il10* was not greater than CD8α-depleted fraction (CD4^+^CD8^−^CD25^+^T cells). The lack of induction of mRNA for perforin and granzyme B suggests these CD8α cells had not acquired a cytotoxic T cells phenotype.

CD8α-enriched fraction of Ts1 cells (CD4^+^CD8α^+^CD25^+^T cells) generated *ex vivo* had enhanced capacity to suppress naïve CD4^+^T cells proliferation in MLC, but this enhanced suppression was not alloantigen-specific. The reason for this lack of specificity of suppression *in vitro* was not examined. From the results of experiments reported here, CD8α expression occurs without proliferation and by exposure of tTreg to rIL-2. It does not require alloantigen stimulation. It appears a significant proportion of tTreg (10% to over 30%) within 3–4 days of exposure to rIL-2 can express CD8α, while retaining expression of CD4, CD25, and Foxp3. These cells also express *Ifngr*, and *l12rb2*. The expression of *irf4* suggests antigen has activated TCR. Stimulation with alloantigen appears to activate alloantigen specific Treg in the CD8α expressing fraction, as seen by the effects of subsequent culture with rIL-12. However, not all CD8α expressing Treg in a Ts1 population have divided or are activated to proliferate by specific alloantigen.

*In vitro* the potency of CD4^+^CD8^+^CD25^+^Foxp3^+^ Treg is non-antigen specific, but *in vivo* where the effects required are over weeks not days, the cells have alloantigen specificity. There is a theoretical advantage in immediate expression of CD8α on tTreg with excess IL-2, which is that they would have enhanced capacity of Treg to recognize antigen presented by class I MHC molecules and produce CD8^+^Treg. One possibility is that the Treg with TCR recognizing antigen on class I MHC are further expanded and could become single positive CD8^+^Treg, much in the way CD4^+^CD8^+^thymocytes mature to single positive CD4^+^ or CD8^+^T cells depending on the affinity of their TCR for Class II and Class I MHC, respectively. Such a transition would allow CD4^+^CD8^−^CD25^+^Foxp3^+^ to produce Treg that can suppress responses to antigen presented by class I MHC molecules. This possibility requires further investigation.

Several groups have identified that the regulatory CD8^+^T cells expresses a homo-dimer of CD8α (46) whereas effector/cytotoxic CD8^+^T cells express the heterodimer CD8αβ (47) in Qa deficient mice (48, 49). CD8αα homodimers can bind to class I MHC and Qa1, and this may promote activation of Treg with TCR recognizing antigen presented by Class I MHC or Qa1. CD8αα^+^ suppressor T cells recognize Qa-1 on activated CD4^+^T cells, B cells and APC and kill these cells to suppress an immune response, reviewed (50). The relationship of these CD8αα^+^Treg to these double positive cells we described, needs to be resolved.

Expression of CD8 by suppressor cells has had a difficult past. The initial description of suppresser cells was CD8α^+^I-J^+^T cells (11). Their existence was questioned when genetic studies failed to detect I-J and suppressor factors used to characterize CD8^+^T suppressor cells (12). More recently a number of CD8^+^Treg have been described (51) and include naturally occurring Treg as well as those induced by an immune response. CD8^+^T suppressor cells have been identified in human transplant recipients (52).

Naturally occurring CD8^+^Treg include CD8αβ^+^T cells that are similar to CD4^+^CD25^+^Foxp3^+^T cells as they express CD25, Foxp3, CLTA4, and GITR and inhibit by cell-to cell contact. These CD8^+^Treg are found in thymus and peripheral lymphoid organs of mice deficient in class II MHC (53) and in human thymus (54).

In rats, CD8^+^T cells with low expression of CD45RC are naturally suppressive of Th1 CD4^+^T cell responses *in vitro* and in GVH responses (55) and mediate allograft tolerance induced by CD40 Ig therapy (16, 18) and anti-CD45RC mAb therapy (56). CD8^+^CD45^lo^Treg suppress by production of IFN-γ and induction of IDO (18).

In the DA rats tolerant to a PVG heart graft, established alloantigen-specific tolerance can be transferred by enriched CD4^+^T cells, whereas CD8^+^T cells enriched by depletion of CD4^+^T cells and B cells do not transfer tolerance (5, 15). As the enrichment of the CD4^+^T cells that transfer tolerance requires depletion of CD8α^+^cells, a double positive (CD4^+^CD8^+^CD25^+^T) cell is not essential to transfer tolerance in the strain combination studied here. We have in preliminary studies shown CD4^+^CD8α^+^CD25^+^Foxp3^+^ T cells are induced *in vivo* during a rejection response and during induction of transplant tolerance. Thus, the early expression of CD8α may be transient in the development pathway of mature alloantigen-specific Treg. This requires further investigation, however.

Collectively, these experiments showed that tTreg culture with rIL-2 alone or with rIL-2 and alloantigen, were induced to express CD8α and some also expressed CD8β. The expression of CD8α was by Treg with greater potency in suppression than the unfractionated Ts1 population induced by culture with alloantigen and rIL-2. CD8α may be of use in enriching the Treg that contain alloantigen-specific Treg for further expansion, including by culture with rIL-12 and specific donor alloantigen.

## Ethics Statement

This study was carried out in accordance with the recommendations of Australian Code for the Care and Use of Animals for Scientific Purposes (NHMRC). The protocol was approved by the Animal Ethics Committee of UNSW, Sydney, NSW, Australia. Rats used in this study were bred and maintained in the animal house, Liverpool Hospital.

## Author Contributions

BH, SH, KP, NV, GT, and NC initiated and designed the research protocols and methods. CR, KP, NV, NC, GT, MN, CW, and PW performed experiments. BH, CR, KP, NV, SH, GT, NC, and PW analyzed the results. BH, NV, SH, AS, and GB wrote the paper.

### Conflict of Interest

The authors declare that the research was conducted in the absence of any commercial or financial relationships that could be construed as a potential conflict of interest.
